# K562 Chronic Myeloid Leukemia Cells as a Dual β3-Expressing Functional Cell Line Model to Investigate the Effects of Combined αIIbβ3 and αvβ3 Antagonism

**DOI:** 10.3390/mps8040073

**Published:** 2025-07-05

**Authors:** Amal A. Elsharif, Laurence H. Patterson, Steven D. Shnyder, Helen M. Sheldrake

**Affiliations:** Institute of Cancer Therapeutics, School of Pharmacy and Medical Sciences, Faculty of Life Sciences, University of Bradford, Bradford BD7 1DP, UKs.d.shnyder@bradford.ac.uk (S.D.S.)

**Keywords:** integrin αvβ3, αIIbβ3, K562, PMA, cell-based functional assay

## Abstract

Several of the integrin family of cell adhesion receptors have been popular targets for the development of anticancer agents, but with little clinical success to date. Cancer cells usually express multiple redundant integrins; one hypothesis for the lack of efficacy of current antagonists is their high selectivity for a single integrin. To address this, we developed a functional dual-β3-expressing cell model to investigate the effects of combined αIIbβ3/αvβ3 antagonism. We established that treating K562 chronic myeloid leukemia cells with 0.04 μM phorbol 12-myristate 13-acetate (PMA) for 40 h significantly upregulates functional αIIbβ3 and αvβ3 integrins. This optimized method provides a reliable platform for adhesion and detachment assays, enabling the characterization of dual integrin targeting strategies. Using this model, we demonstrate that combining αIIbβ3 and αvβ3 antagonists (GR144053 and cRGDfV) synergistically enhances inhibition of cell adhesion and promotes cell detachment compared to single-agent treatments. Our findings establish a reproducible approach for studying dual β3 integrin targeting, which can be used to investigate potential strategies for overcoming integrin redundancy in cancer therapeutics.

## 1. Introduction

The integrin adhesion receptors are a family of 24 non-covalent heterodimers, each containing an α and a β subunit. The integrin family is divided into subfamilies that recognize common extracellular matrix (ECM) ligands. Cells adhere to the extracellular matrix through integrin heterodimers that bridge the cell membrane, connecting the ECM to the actin cytoskeleton [1].

Several integrins, notably those recognizing the Arg-Gly-Asp (RGD) tripeptide sequence, have been strongly associated with the spread of cancer and poor prognosis [2,3]. This includes the two β3 subunit integrins, αIIbβ3 and αvβ3. αvβ3 is highly expressed in activated endothelial cells and plays a fundamental role in cell proliferation and migration, resulting in angiogenesis [4]. It is upregulated in several cancers, including melanoma and glioblastoma [5,6,7,8]. αIIbβ3 is the major integrin receptor expressed on platelets, where it is responsible for crosslinking with fibrinogen during the clotting process. It is also expressed in some types of tumors, including melanoma and prostate adenocarcinoma. Tumoral αIIbβ3 promotes tumor growth in vivo through supporting cell survival and angiogenesis [9] and tumor invasiveness [10,11]. Interactions between platelets and cancer cells, supported by β3 integrins on both cell types, are essential to metastatic dissemination and therefore constitute a promising cancer target [12,13,14].

Despite the significant interest in αvβ3 (anti-angiogenic) and αIIbβ3 (anti-thrombotic) antagonists, relatively few dual β3-targeted agents have been developed. The clinically used anti-αIIbβ3 antibody Abciximab has relatively high anti-αvβ3 activity [15], but other dual-targeted anti-thrombotic agents have not progressed past preclinical development [16,17,18]. Repurposing Abciximab for indications outside of acute cardiology has not been investigated.

In cancer, interactions between integrins and the extracellular matrix lead to changes in the identity and amount of integrin proteins on the cells’ surface. Changes in the integrin proteins expressed and redundancy of integrin functions allow cells to evade the effects of selective anti-integrin agents [9,19,20]. We hypothesize that dual-targeted β3 antagonists will have significantly enhanced efficacy compared to selective αvβ3 antagonists. They will be able to block integrin redundancy in dual β3-expressing tumors as well as bidirectional targeting of cancer cell interactions with platelet αIIbβ3 and endothelial cell αvβ3 integrin.

The ECM makes a significant contribution to cancer progression and metastasis [21]. Interactions between tumor cells and ECM proteins trigger signaling pathways leading to cell survival and proliferation. The presence of specific proteins such as osteopontin promotes site-specific metastasis [22]. The presence of cancer-associated fibroblasts and changes in ECM stiffness also promote cell proliferation and decrease response to anticancer therapies [23,24]. In leukemia, the bone marrow microenvironment supports the survival of stem cells and thereby the persistence of minimal residual disease [25]. Therefore, tumor-ECM interactions have been proposed as a therapeutic target (reviewed in [26,27,28]). In chronic myelocytic leukemia (CML), changes in integrin-mediated interactions between leukemia cells and fibronectin allow the circulation of malignant cells [29]. Binding of K562 cells to RGD-containing-ligands triggers resistance to clinically used drugs [30].

αIIbβ3 and αvβ3 play significant roles in tumor-microenvironment interactions in leukemias [31]. αvβ3-osteopontin binding promotes treatment resistance [32], and αIIbβ3 binding fibrinogen and fibronectin [33] could have a similar effect. The K562 leukemia cell line has previously been used as a model to investigate the effect of integrin agonists on α5β1-mediated adhesion [34]. In this model, it was noted that αIIbβ3 also contributed to the cells’ adhesion to fibronectin [34].

There is a lack of dual β3-expressing cell line models to investigate anticancer therapeutics. Studies typically use cell-free assays or cell lines expressing individual integrins [35,36]. While transfection has previously been used to express the αv integrin subunit in K562 [37], this method is not always robust. Another method that could be considered is to induce expression in the cells. PMA is a potent inducer of the megakaryocytic phenotype in leukemic cell lines. This megakaryocytic phenotype is marked by upregulation of αIIb [38,39,40,41] and β3 integrin subunit expression [38,42]. Furthermore, the αv integrin subunit may also be upregulated by PMA treatment [43]. Therefore, we hypothesized that PMA-treated K562 cells can provide a useful model for initial assessment of the effects of dual β3 integrin antagonism. Here, we report the validation of PMA-treated K562 as a functional model for investigating β3-targeted agents and demonstrate the effects of selective β3 antagonists used alone and in combination.

## 2. Materials and Methods

### 2.1. Cell Culture and Reagents

The human chronic myeloid leukemia cell line K562, which grows in suspension, was obtained from the American Type Culture Collection (Manassus, VA, USA). Cells were maintained in RPMI 1640 containing 10% fetal bovine serum (FBS), 200 mM L-glutamine, and 100 mM sodium pyruvate. GR144053 was sourced from Tocris Bioscience (Abingdon, UK), and cRGDfV from Peptanova (Heidelberg, Germany). Monoclonal rabbit anti-αIIb (EPR4330) and polyclonal rabbit anti-β5 (ab15459) antibodies were from Abcam (Cambridge, UK). Polyclonal rabbit anti-αv (Q-20) and monoclonal mouse anti-β3 (B-7) and anti-α5 (C-9) antibodies were from Santa Cruz Biotechnology (Heidelberg, Germany). Donkey anti-rabbit IgG H&L and goat anti-mouse IgG H&L (Alexa Fluor^®^ 488) were from Abcam. All other reagents and supplies were purchased from Sigma-Aldrich (Gillingham, UK), unless otherwise specified.

### 2.2. Immunofluorescence Analysis

PMA working solutions were prepared by diluting a 10 mM stock solution in DMSO with RPMI medium to the required concentrations. K562 cells were treated with PMA at concentrations of 0.04, 0.08, or 0.1 μM. To investigate the effect of PMA over time, each concentration was incubated for 24, 48, and 72 h. After incubation, a cell suspension was prepared, and 5 × 10^5^ cells/mL were seeded on coverslips pre-coated with poly-L-lysine solution (0.1%) in 6-well plates and incubated in 5% CO_2_ at 37 °C overnight. Cells were fixed with 4% paraformaldehyde, blocked with 5% bovine serum albumin (BSA)/phosphate-buffered saline (PBS) for 1 hour at room temperature, and incubated with primary antibody at 4 °C overnight. The cells were washed with PBS for 3 × 5 min at 37 °C. Secondary antibody was added and incubated for one hour in a dark place at room temperature. The coverslips were washed again and then mounted with a drop of Vectashield anti-fade mounting medium with DAPI stain (Vector Laboratories, 2BScientific, Kirklington, UK). For sample analysis, fluorescent images were captured using a Leica DM2000 microscope (Wetzlar, Germany) and then processed using the Leica Application Suite v4.0. Method developed from [44]

### 2.3. Flow Cytometry Analysis

Flow cytometric analysis was carried out as described previously [45]. K562 cells, previously treated with high (0.1 µM) or low (0.04 µM) concentrations of PMA, or untreated as a control, were harvested and washed twice in PBS. The cells were then fixed with 4% PFA and incubated with the relevant primary antibody for 45 min at 4 °C. After washing in cold PBS, cells were incubated with a fluorochrome-labeled (FITC or TRITC dye) secondary antibody for 30 min at 4 °C. Cells were washed, re-suspended in cold PBS, and kept on ice until analyzed by flow cytometry (FACSCalibur^TM^, Becton Dickinson, NJ, USA).

### 2.4. MTT (3-(4,5-Dimethylthiazol-2-yl)-2,5-diphenyl Tetrazolium Bromide) Assay

Cell viability was measured using the MTT assay as described previously [46]. Briefly, cells were cultured in 96-well plates containing a total volume of 180 µL/well. 20 μL of filter-sterilised MTT per well (5 mg/mL in distilled water filtered through a 0.2 µm filter and stored for a maximum of 4–6 weeks at 4 °C) was added and incubated for 4 h. Following incubation, the media was gently removed, and 150 μL of sterile DMSO was added and mixed with the blue formazan crystals. Finally, the absorbance of plates was read at 540 nm. IC_50_ was calculated from the dose response curves as the concentration which reduced viability to 50% compared to the untreated control cells

### 2.5. Optimisation of Cell Adhesion Assay

96-well plates (treated microplate, Costar, Corning, NY, USA) were coated with 100 µL/well of fibrinogen (2 µg/mL) and incubated overnight at 4 °C. Fibrinogen was removed and wells washed three times with PBS. The wells were blocked with 3% BSA in PBS (100 μL/well) for 1–2 h at 37 °C. K562 cells were stimulated by incubation in RPMI medium (with additives described in Section 2.1) containing PMA (0.04 μM) for 40 h at 37 °C. The medium was removed and replaced by RPMI containing 200 mM L-glutamine, and 100 mM sodium pyruvate without FBS. Six different concentrations, 5 × 10^4^–5 × 10^5^ cells/mL, of K562 cells were added to the fibrinogen-coated plates and incubated at 37 °C for one hour. Cell adhesion was compared to PMA-untreated cells. The supernatant was removed and the wells were washed twice with PBS to remove non-adherent cells. 180 μL of complete medium was added to each well and incubated overnight at 37 °C. Finally, cell binding was measured by MTT assay as described above. Methods were adapted from [36].

### 2.6. Adhesion and Detachment Assays to Investigate Integrin Targeted Agents

96-well plates (treated microplate, Costar, Corning, NY, USA) were coated with 100 µL/well of fibrinogen (2 µg/mL) and incubated overnight at 4 °C. Fibrinogen was removed and wells washed three times with PBS. The wells were blocked with 3% BSA in PBS (100 μL/well) for 1–2 h at 37 °C. K562 cells were stimulated by incubation in RPMI medium (with additives described in Section 2.1) containing PMA (0.04 μM) for 40 h. The medium was removed and replaced by RPMI containing 200 mM L-glutamine, and 100 mM sodium pyruvate without FBS. Cells were then treated with different concentrations of GR144053 and cRGDfV for 4 h on a rotary shaker at room temperature. −PMA cells were treated identically with the omission of the PMA stimulation step. Control cells were treated identically without integrin inhibitors. After the 4 hour incubation, cells were added to the fibrinogen-coated plates and incubated at 37 °C for one hour. The wells were washed twice with PBS and 180 μL of complete medium was added to each well and incubated overnight at 37 °C. Cell adhesion was measured by MTT assay as described above.

For the detachment assay, 96-well plates (non-treated microplate, Corning) were precoated with fibrinogen or coated with 100 µL/well of 5% BSA in PBS as a blocking agent (control for non-specific adhesion). ±PMA treated K562 cells were seeded in complete medium and incubated overnight at 37 °C and then treated with different concentrations of GR144053 and cRGDfV for 6 h at 37 °C. Control cells were treated identically without integrin inhibitors. After each incubation period, the supernatant was removed and the wells washed gently with PBS. The remaining adherent cells were quantified by MTT assay and percent detachment calculated as (100—remaining adherent cells). IC_50_ was calculated from the dose response curves as the concentration which inhibited adhesion or induced detachment by 50% compared to control (no integrin inhibitor) cells. Methods were adapted from [36].

### 2.7. Statistical Analysis

Student’s *t*-test (two tailed) was utilised for statistical analysis of functional assay using Excel software. Results were considered statistically significant for *p* < 0.05 and highly significant for *p* < 0.01. CompuSyn version 1.0 (Combosyn Inc. Paramus, NJ, USA) was used to calculate the combination index (CI) in drug combinations [47]. CI < 1, CI = 1 and CI > 1 indicate synergistic, additive, and antagonistic effects, respectively.

## 3. Results

### 3.1. Effect of PMA Treatment on Integrin Expression in K562 Cells

**Figure 1:**
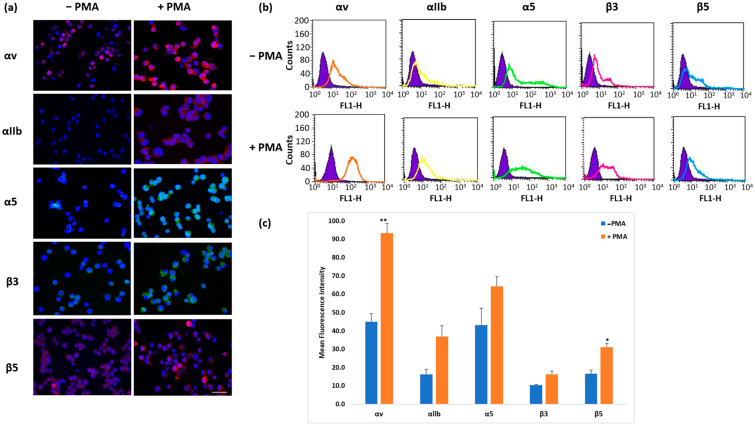
Effect of PMA treatment on αv, αIIb, α5, β3, and β5 integrin subunit expression in K562 cells. K562 cells were treated with PMA (0.1 μM) for 24 h. The expression of αv, αIIb, α5, β3, and β5 integrin subunits was detected by immunofluorescence (**a**) and flow cytometry (**b**) with anti-αv (Q20), anti-αIIb (EPR4330), anti-α5 (C-9), anti-β3 (B-7), or anti-β5 (ab15459) integrin antibodies. Scale bar, 50 μm. Blue-fluorescent DAPI is nuclear staining, and colors (green and red) represent integrin expression detected by a fluorophore (TRITC or FITC). The analysis of data from B (**c**) is shown as mean ± standard error of 3 independent repeats. Statistical significance: * *p* < 0.05, ** *p* < 0.01.

The expression of a range of RGD-recognizing integrin subunits in K562 cells treated with PMA (0.1 μM) for 24 h was higher than in untreated cells (Figure 1). The largest increases were observed for αv and αIIb, with smaller effects on β3 and β5 subunit levels. Increases in αv and β5 were statistically significant (*p* < 0.05); the increase in αIIb did not reach statistical significance (*p* 0.059). All integrin subunit screening results using fluorophore-labeled antibodies showed that K562 cells expressed these integrin subunits cytoplasmically. However, flow cytometry demonstrated cell surface expression, indicating the presence of functional integrin heterodimers, thus confirming the suitability of the cell line model for use in functional assays.

#### 3.1.1. Effect of PMA on K562 Cell Viability

**Figure 2:**
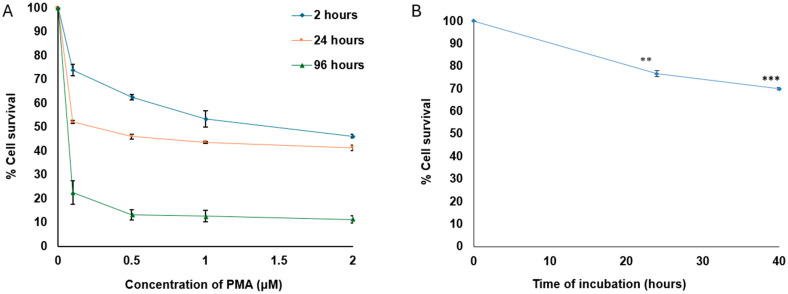
Effect of PMA on K562 cell viability determined using the MTT assay. (**A**) Cell survival curves, which show a similar moderate effect at all doses evaluated for both 2- and 24-hour incubations, with approximately 50% cell survival after 24 h. However, when cells are exposed to PMA for 96 h, a much greater cytotoxic effect is seen at all doses evaluated. (**B**) Cell survival curve demonstrating that incubating with a lower dose of PMA (0.04 µM) can maintain good cell viability while still inducing αIIb expression (see Figure 3). Data are shown as the mean ± standard error of 3 independent results. Statistical significance: ** *p* < 0.01, *** *p* < 0.001.

**Figure 3:**
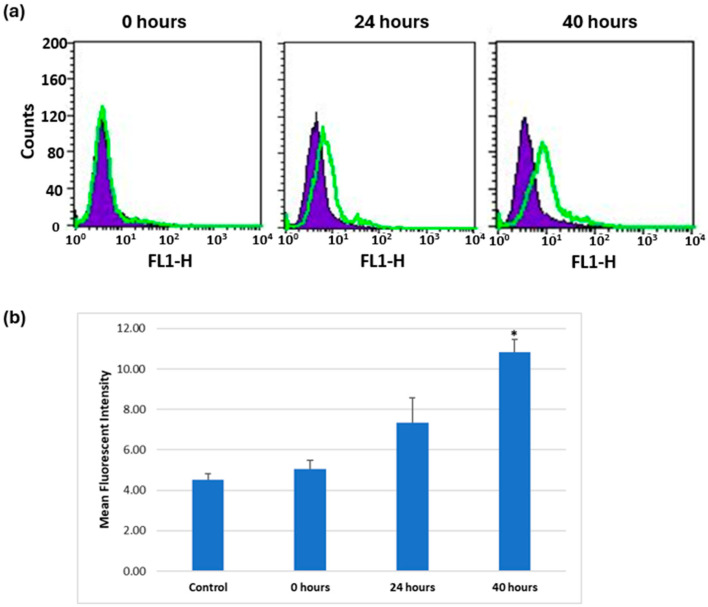
Expression of αIIb in K562 cells treated with 0.04 µM of PMA at three time points. (**a**) αIIb expression detected by anti-αIIb (EPR4330) is shown in green compared to negative control in purple. (**b**) Analysis of flow cytometry data using mean fluorescence intensity. Data are shown as mean ± standard error of 3 independent results. Statistical significance: * *p* < 0.05.

The effect of PMA on cell viability was determined to assist selection of a concentration of PMA and incubation time that would stimulate integrin expression with minimal effect on cell viability. PMA concentrations in the range of 0.04–2 μM were investigated (Figure 2). PMA affected cell viability in a dose-dependent manner. At an incubation time of 2 h, in which no cell division would be anticipated, concentrations of PMA up to 0.5 µM had little effect on cell viability. Increasing the PMA incubation time to 24 h had a moderate effect on cell viability, whereas exposure to PMA for 96 h was more cytotoxic at all concentrations. Treatment of cells with approximately ten-fold lower concentration (0.04 µM PMA) for 24 and 40 h (Figure 2B) upregulated expression of αIIb (Figure 3) with a tolerable effect on cell survival, suggesting this concentration can be used for functional assays.

#### 3.1.2. Induction of αIIb Expression over Time in K562 Cells Treated with 0.04 µM PMA

To allow a broader time window to assess the effects of treatment, a lower concentration of PMA (0.04 µM) was administered, and αIIb expression was evaluated at different time points. As seen in Figure 3, a statistically significant increase in αIIb expression was seen after 40 h treatment. A 40-hour pretreatment period was therefore chosen to induce integrin expression in cells for use in functional assays.

### 3.2. Evaluation of K562 Cell Adhesion to Fibrinogen

**Figure 4:**
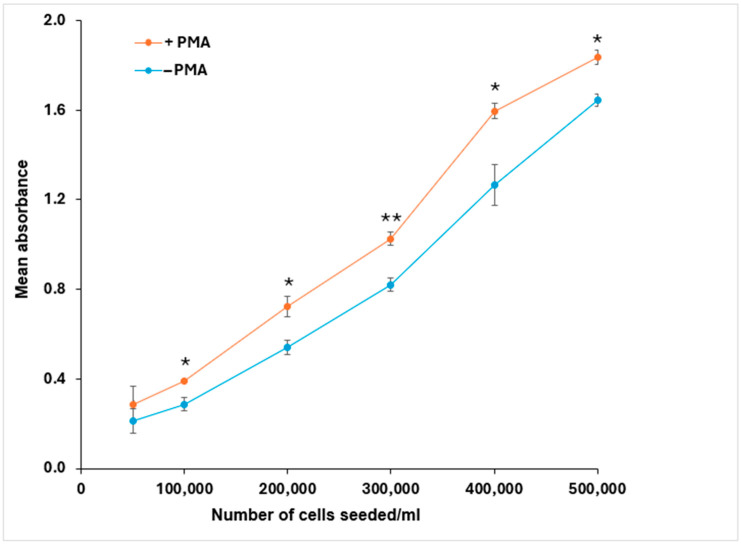
PMA increases K562 cell binding to fibrinogen. K562 cells (±0.04 μM PMA for 40 h) were allowed to adhere to a plate coated with 2 µg/mL of fibrinogen for 4 h at 37 °C. Cell binding significantly increased with PMA induction at all cell seeding densities over 1 × 10^5^ cells/mL. Results shown are the mean ± standard error of 3 independent experiments. Statistical significance: * *p* < 0.05, ** *p* < 0.01.

Adhesion assay methods depend on the ability of integrin receptors on the cell surface to interact with extracellular matrix proteins on a coated plate. Fibrinogen was chosen as an RGD-containing ECM ligand for both β3 integrins while not being a ligand for α5β1 or αvβ5 [48]. To optimize the cell number, six different concentrations of cells (5 × 10^4^–5 × 10^5^ cells/mL) were treated with 0.04 μM PMA in FCS-free medium, and adhesion was compared to the same numbers of untreated cells. FCS-free medium is required in adhesion assays to avoid competing binding to RGD-containing proteins in serum. Treatment with PMA increased cell binding to fibrinogen (Figure 4). 500,000 cells/mL was chosen as the best cell density for further experiments.

### 3.3. β3 Antagonists Inhibit K562 Adhesion to Fibrinogen

**Figure 5:**
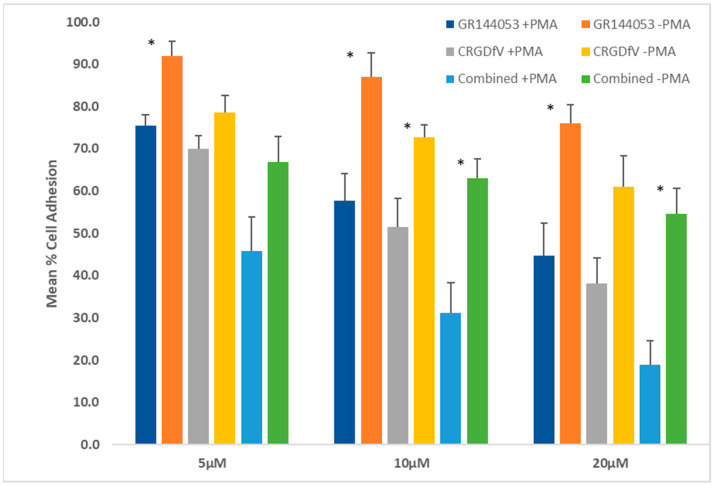
Effects of GR144053 and cRGDfV on K562 cell adhesion to fibrinogen. The adhesion of K562 cells to fibrinogen was assessed under two conditions: untreated or pretreated with 0.04 µM PMA for 40 h. Cells were treated with the compounds for 4 h, and the percentage of adherent cells was quantified colorimetrically relative to the untreated no-inhibitor controls (100% adhesion). Data represent the mean ± standard error of three independent experiments. Statistical significance: * *p* < 0.05.

The effect of selective antagonists targeting αvβ3 (cRGDfV) and αIIbβ3 (GR144053) was investigated using K562 cells with induced integrin expression in a fibrinogen adhesion assay (Figure 5). In untreated K562 cells, adhesion was partially dependent on αvβ3 integrin, whereas inhibiting αIIbβ3 had little effect. PMA-treated cells showed an increased dependence on both β3 integrins, as 20 µM of combined antagonists significantly blocked cell adhesion. Potential interaction between cRGDfV and GR144053 was evaluated by combination index (CI) analysis [47]. A synergistic effect was observed at all concentrations tested (Table 1) and IC_50_ values of cell adhesion (Table 2).

### 3.4. β3 Antagonists Promote Cell Detachment from Fibrinogen

**Figure 6:**
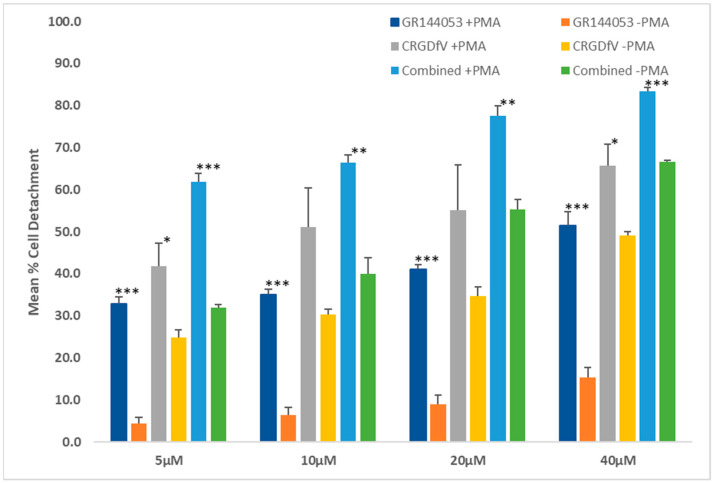
Effects of cRGDfV and GR144053 on K562 cell detachment. Cells (pretreated with ±0.04 µM PMA for 40 h) were treated with the compounds for 6 h. The percentage of adherent cells remaining was quantified colorimetrically relative to the untreated no-inhibitor controls, and percentage detachment was calculated as (100 − adherent). Treatment with GR144053 alone or combining both agents significantly increased cell detachment following PMA pretreatment. Data represent the mean ± standard error of three independent experiments. Statistical significance: * *p* < 0.05, ** *p* < 0.01, *** *p* < 0.001.

The ability to disrupt pre-existing integrin-ligand interactions is a distinct property from preventing the formation of new adhesive interactions. Therefore, we next investigated the effects of combined β3 antagonism on K562 detachment from fibrinogen (Figure 6). β3 antagonists cRGDfV and GR144053 had limited ability to induce detachment when used as single agents. Blocking αvβ3 was more effective than blocking αIIβ3. Both inhibitors showed increased effects in PMA-pretreated cells, with the effect of GR144053 being more markedly increased (at least three-fold compared to untreated cells). Synergistic effects were observed with all combinations of antagonists tested (Table 1).

## 4. Discussion

Blocking integrin-extracellular matrix interactions is an important strategy in inhibiting angiogenesis and cancer metastasis. While there is strong preclinical evidence that interfering with β3 function reduces tumor growth and dissemination, e.g., [49,50,51], αvβ3-targeted agents have not lived up to this potential in the clinic [52]. Inhibition of cancer cell and endothelial cell αvβ3, or platelet αIIbβ3, can reduce tumor growth in vivo [53,54]. Targeting both receptors simultaneously should increase anticancer effects by inhibiting more types of β3-mediated interaction and effectively targeting dual β3-expressing tumor cells. αIIbβ3 expression may be acquired as part of the tumorigenesis process or by transfer of integrins from platelet-derived microparticles [55] and will allow cells to evade the effects of selective αvβ3-targeted agents.

The main purpose of this study was to investigate a potential dual αIIbβ3 and αvβ3 expressing cell-based model. PMA treatment of K562 cells is known to increase their expression of αIIbβ3 [38,39,40,41] and αvβ3 [43] integrins and promote interaction with ECM fibronectin and vitronectin [56,57,58]. We therefore investigated the utility of PMA-treated K562 cells as a readily available and reliable dual αIIbβ3- and αvβ3-expressing functional model.

Incubation with PMA significantly increased K562 cell surface expression of αIIb in a time- and dose-dependent manner and also enhanced the expression of other RGD-binding integrin subunits (αv, α5, β3, and β5), particularly αv. Flow cytometry confirmed that these subunits were present on the cell surface, indicating that expression corresponded to the formation of functional heterodimers (Figure 1 and Figure 3). The increase in fluorescence intensity used to detect integrin subunit expression could result either from an increase in the amount of subunit protein expressed by cells or an increase in the number of cells expressing the subunit. Further work is required to distinguish these two possibilities. It should be noted that baseline expression of αIIbβ3 in K562 detected by other methods is negligible [41].

PMA-treated K562 cells showed increased adhesion to fibrinogen compared with unstimulated cells, confirming that the integrin receptors induced by PMA treatment were functional for cell-ECM interactions (Figure 4). For adhesion and detachment assays, we used 0.04 µM PMA for 40 h, as this protocol provided significant expression of αIIb and a cell density suitable for reproducible measurements.

We chose the established integrin antagonists cRGDfV and GR144053 to investigate the effects of selective and combined inhibition of αvβ3 and αIIbβ3 on K562 cells’ interactions with fibrinogen, a ligand of both receptors (Figure 5 and Figure 6). Blocking individual integrins by either GR144053 or cRGDfV reduced the cells’ ability to adhere to fibrinogen, and induced detachment of adherent cells. Greater effects were observed in PMA-treated cells, which express higher levels of both β3 integrins. Combining the two integrin antagonists caused a synergistic increase in inhibition of adhesion and induction of detachment, although it did not completely prevent K562 interactions with fibrinogen. A limitation of all cell-based investigations of integrin antagonism is that cells express multiple different integrins, some of which have overlapping functions. According to Baiula et al., some K562 cell adhesion is mediated by α5β1 [59], however their studies used fibronectin as the ligand which has a much higher affinity for α5β1 than the fibrinogen in our work. Judicious choice of ECM ligand is essential in integrin assays.

The ability of integrin antagonists to cause cell detachment is important in reducing metastasis by limiting the ability of cells to form tumors after they have already reached and adhered to a secondary site. Previous studies on cell detachment have given contradictory results. Blocking the activity of αvβ3 and αvβ5 integrins has been shown to induce detachment of cancer cell lines [60,61], but other studies, using isolated proteins, have shown antagonists cannot disrupt integrin-ligand complexes once formed [62]. This work provides further support for the ability of integrin antagonists to reverse existing integrin-mediated cell-matrix interactions.

The effect of dual β3 inhibition has been previously investigated using Abciximab-related antibodies in vivo [53,54,63], and combinations of small molecules in vitro [64]. In vivo models of dual antagonism are complex and require the use of rats for studies of inhibitory antibodies. In vitro models are preferable to comply with animal welfare and 3Rs aspirations. Only one in vitro test of dual β3 antagonism has been reported; combination of the two antagonists lamifiban (anti-αIIbβ3) and SB273005 (anti-αvβ3) synergistically increased the inhibition of adhesion of MDA-MB-231 cells to HUVEC ECM under blood flow conditions [64]. Our data supports this study in indicating that targeting αIIbβ3 or αvβ3 alone is insufficient to fully inhibit β3-mediated adhesion in systems where both integrins are present.

A limitation of the PMA-induced model of integrin expression reported here is that it has only been used in vitro and would be challenging to translate in vivo. There is a lack of pharmacokinetic information on PMA rendering dose-finding challenging. PMA induces differentiation so can change the clinical course of leukemia [65,66] which could become a confounding factor in in vivo studies of other compounds. A transfected cell line would provide an alternative approach in vivo. There are multiple literature reports of transfected cell lines expressing a single integrin. For example, GlaxoSmithKline have developed K562 cell lines stably transfected to express αv subfamily integrins [67], which have been used in in vitro adhesion assays [68]. αvβ3-transfected cells have been used in vivo to investigate the effects of the integrin on tumor progression, e.g., [69]. Transfection to express αIIbβ3 is less common as αIIbβ3 is predominantly expressed on platelets so its function is assessable by readily available ex vivo aggregation and in vivo bleeding time assays. A dual αIIbβ3/αvβ3 expressing melanoma cell line model has been reported and its tumor growth characterised in vivo [9], however it has not been used with any integrin-targeted therapeutics. Transfecting K562 to express αIIb, αv and β3 subunits could yield a leukemia model suitable for use both in vitro and in vivo investigation of hit and lead compounds. In comparison to transfection, the reported model provides a readily available and inexpensive method for initial investigation of compounds in early drug discovery.

## 5. Conclusions

In conclusion, this study highlights the development of a method for evaluating the dual inhibition of αIIbβ3 and αvβ3 integrins to disrupt cancer cell adhesion and promote detachment from the extracellular matrix. Using PMA to induce integrin expression in K562 cells, we demonstrated that dual targeting of both β3 integrins significantly reduced adhesion and increased detachment efficacy compared to single integrin inhibition. These findings support the hypothesis that dual β3 integrin inhibition may offer an effective approach to reduce metastasis. This is the first study to use a dual β3-expressing cell line to investigate the effects of integrin antagonists. The method could be expanded by integrating additional integrin-mediated pathways to broaden its application in screening integrin-targeted cancer therapies.

## Figures and Tables

**Table 1:** Combination index (CI) values for the effect of GR144053 and cRGDfV on K562 (±0.04 µM PMA) binding to fibrinogen in adhesion and detachment assays. CI < 0.1 very strong synergism, 0.1–0.3 strong synergism, 0.3–0.7 synergism, 0.7–0.85 moderate synergism, 0.85–0.90 slight synergism, and 0.90–1.10 additive.

K562	cRGDfV (μM)	GR144053 (μM)	Total Dose (μM)	Adhesion CI Value	Detachment CI Value
+PMA	5.0	5.0	10.0	0.33	0.20
	10.0	10.0	20.0	0.34	0.12
	20.0	20.0	40.0	0.34	0.17
	40.0	40.0	80.0	-	0.16
−PMA	5.0	5.0	10.0	0.25	0.18
	10.0	10.0	20.0	0.42	0.13
	20.0	20.0	40.0	0.51	0.17
	40.0	40.0	80.0	-	0.19

**Table 2:** IC_50_ values for inhibition of cell adhesion by β3 integrin antagonists.

Antagonists	K562 − PMA IC_50_ ± SD (μM)	K562 + PMA IC_50_ ± SD (μM)
cRGDfV	20	12.6 ± 6.4
GR144053	>20	14.1 ± 5.2
GR144053 and cRGDfV	17.7 ± 3.2	5.9 ± 1

## Data Availability

The original contributions presented in this study are included in the article. Further inquiries can be directed to the corresponding author(s).
